# The emerging role of next-generation sequencing in minimal residual disease assessment in acute lymphoblastic leukemia: a systematic review of current literature

**DOI:** 10.3389/fmed.2025.1570041

**Published:** 2025-04-22

**Authors:** Andreea-Iulia Ștefan, Letiția-Elena Radu, Dumitru Jardan, Anca Coliță

**Affiliations:** ^1^Department of Medicine, University of Medicine and Pharmacy “Carol Davila”, Bucharest, Romania; ^2^Department of Pediatrics, Fundeni Clinical Institute, Bucharest, Romania; ^3^Molecular biology laboratory, Medlife Clinic, Romania

**Keywords:** acute lymphoblastic leukemia, minimal residual disease, next-generation sequencing, risk stratification, event-free survival, relapse prediction

## Abstract

**Background:**

Minimal residual disease (MRD) is a critical prognostic marker in acute lymphoblastic leukemia (ALL). The well studied and used MRD detection methods, multiparametric flow cytometry (MFC) and real-time quantitative polymerase chain reaction (qRT-PCR) for fusion genes and receptor gene rearrangements have significantly improved risk stratification, but have limitations in sensitivity and applicability. Next-generation sequencing (NGS) has emerged as a promising approach for MRD assessment, offering better sensitivity and the ability to track clonal evolution.

**Objectives:**

This systematic review evaluates the clinical utility and prognostic value of NGS for MRD detection in ALL, comparing its performance with conventional methods and exploring its potential role in therapeutic guidance.

**Methods:**

A comprehensive literature search was conducted across PubMed and Web of Science following PRISMA guidelines. Studies were included if they assessed MRD using NGS in ALL patients and provided data on sensitivity and prognostic value. Comparative analyses with MFC or qRT-PCR were considered. Data on end-of-induction MRD values, event-free survival (EFS), and overall survival (OS) were extracted.

**Results:**

Thirteen studies met the inclusion criteria. NGS demonstrated superior sensitivity in detecting MRD-positive cases compared to MFC in patients classified as MRD-negative. Higher correlation was observed in MRD-positive cases than in MRD-negative cases. NGS-based MRD stratification correlated strongly with clinical outcomes, with patients achieving NGS-MRD negativity exhibiting superior EFS and OS rates. Additionally, NGS was highly predictive of relapse following hematopoietic stem cell transplantation and CAR-T cell therapy. The IGH rearrangements as the primary marker in NGS panels has demonstrated good prognostic value in B-ALL.

**Conclusion:**

NGS represents a transformative tool for MRD monitoring in ALL, offering enhanced sensitivity and prognostic accuracy. Challenges such as high costs, complex bioinformatics analysis and the need for standardization remain. While its integration into clinical practice holds significant promise, further research is needed to establish standardized protocols, cost-effectiveness, and its optimal role in treatment decision-making. The combination of NGS with MFC may provide complementary advantages.

## 1 Introduction

*Acute lymphoblastic leukemia (ALL)* is the most common type of cancer in children, with a peak prevalence between the ages of 2 and 5 years and a male predominance. Overall survival in this disease has improved dramatically over the last 40 years, reaching over 90% in the pediatric population. In adults, ALL is not a commonly found cancer, accounting for less than 1% of all cancers (1). Its long-term survival rate is inferior than in children, being one of the most challenging cancers in adults. The significant increase in ALL survival rates is due to the introduction of patient risk stratification based on prognostic factors, which allowed for the adjustment of treatment intensity and duration according to each patient’s individual characteristics (2–4). In pediatric patients, the most common genetic abnormalities are high hyperdiploidy (>50 chromosomes) and ETV6::RUNX1 fusion gene. Both of them offer a favorable prognosis and are included in the stratification process (5, 6). Multiple other genetic mutations and chromosomal abnormalities are recognized as key prognostic indicators for high-risk disease, such as hypodiploidy, KMT2A rearrangements, TCF3::HLF fusion, and BCR::ABL1 positive ALL (6). Despite significant advancements in treatment protocols, relapse remains a major obstacle to improving long-term survival outcomes.

*Minimal residual disease (MRD)*, defined as the presence of leukemic cells below the detection threshold of conventional testing methods, is a critical prognostic marker in ALL. The early response to induction chemotherapy is the most important independent prognostic factor in ALL (7, 8). Traditionally, multiparametric flow cytometry (MFC) and real-time quantitative polymerase chain reaction (qRT-PCR) are the gold standards for MRD detection, offering a high degree of sensitivity (10^-4^) and specificity. However, these techniques have limitations (Table 1). qRT-PCR for receptor gene rearrangements is a laborious, time consuming method. The analyzing and primer selection can take up to 3–4 weeks. Moreover, a large amount of DNA is required at diagnosis and changes in the initial clone or the emergence of new clones during treatment can lead to false-negative results. The detection of fusion gene transcripts using qRT-PCR has limited applicability, as over 50% of cases do not have detectable fusion genes of those tested standardly at diagnosis. While it offers the advantage of being stable throughout treatment and requires only a single set of primers, its accuracy is affected by variability in the number of RNA transcripts per leukemic cell, both among different patients and within the same clone (9). MFC has proven to be a valuable tool for monitoring MRD in ALL. It is a fast and widely applicable technique, capable of being used in all cases. However, it does come with several limitations. One major issue is its reliance on the skill and experience of the technicians and analysts. Additionally, antigen expression can shift during treatment, making it harder to detect leukemic clones. Furthermore, emerging immunotherapeutic strategies, such as cellular therapies and monoclonal antibodies targeting antigens like CD19 (Blinatumomab) or CD22 (Inotuzumab ozogamicin), present new challenges for accurately identifying specific leukemic clones using MFC (10).

**Table 1:** Advantages and disadvantages of current methods for measuring minimal residual disease.

Method	Advantages ↑	Disadvantages ↓
Multiparametric flow cytometry	Fast Widely applicable Relatively cheap Standardized Non-viable cells excluded from the analysis	Modulation of antigen expression over the course of the disease can lead to false negative results Reliance on the skill and experience of the technicians and analysts Limited sensitivity using <8 colors Influenced by immunotherapy
qRT-PCR for fusion genes	Very good sensitivity Relatively simple No patient-specific primers necessary	Limited applicability (<50% have fusion genes) Cannot detect subclones/clonal evolution
qRT-PCR for receptor gene rearrangements IG/TCR	High sensitivity Thoroughly standardized within EuroMRD Consortium	Needs patient specific primers Time consuming Costly Not able to define accurately the amount of residual disease in cases where the disease burden is very low
NGS for receptor gene rearrangements IG/TCR	High sensitivity (down to 10^−6^) Universal primer sets used in all patients Can detect subclones/clonal evolution and immune repertorium	Standardization in progress within EuroClonality- NGS Consortium group High degree of experience in bioinformatics Costly

IG, immunoglobulin; NGS, next generation sequencing; TCR, T-cell receptor; qRT-PCR, real-time quantitative polymerase chain reaction. Adapted from Kotrova et al. (9).

Next *Generation Sequencing (NGS)* has revolutionized the landscape of MRD detection. Unlike MFC and qRT-PCR, NGS provides a comprehensive method to sequence entire regions of DNA or RNA, focusing on sequencing immunoglobulin (Ig) and T-cell receptor (TCR) gene rearrangements to offer a unique molecular fingerprint for each leukemic clone. NGS allows for the precise identification of minimal residual disease, even at very low levels (10^-6^). Moreover, NGS can detect clonal evolution, identifying changes in the genetic landscape of leukemic cells. This ability to monitor disease dynamics in real time makes NGS a valuable tool in guiding treatment decisions, particularly in high-risk cases (11, 12). As NGS continues to be integrated into clinical practice, its potential to redefine MRD monitoring and improve patient outcomes in ALL becomes increasingly apparent. However, challenges remain, like the cost of NGS technology, the need for standardized protocols and the interpretation of complex sequencing data. The Euro Clonality-NGS study group is working to develop standardized guidelines for data analysis and interpretation (13).

Given the increase of NGS in clinical practice, this systematic review aims to evaluate the latest literature on its role in MRD assessment for ALL. Specifically, we seek to assess the clinical utility and prognostic value of NGS. By systematically analyzing recent studies, this review will provide an overview of the current state of NGS-based MRD detection and highlight areas for future research.

## 2 Methodology

### 2.1 Search strategy

This systematic review was conducted following the PRISMA (Preferred Reporting Items for Systematic Reviews and Meta-Analyses guidelines) guidelines to evaluate the role of NGS in the assessment of MRD in ALL. Our review aims to provide a comprehensive analysis of recent studies involving NGS for MRD detection, compared with traditional methods and correlation with relapse and overall survival. A comprehensive search was conducted across major databases (PubMed and Web of Science). The search strategy was developed using a combination of keywords related to “acute lymphoblastic leukemia”, “minimal residual disease” and “next-generation sequencing”.

### 2.2 Eligibility criteria

Eligible studies were those involving patients diagnosed with ALL who were assessed for MRD using NGS and included a minimum of 10 patients. We included studies that either compared NGS with other MRD detection methods, such as MFC, qRT-PCR or reported on NGS alone. The studies needed to provide data on the sensitivity, prognostic value or clinical utility of NGS for MRD detection. We considered a range of study designs, randomized controlled trials, cohort studies, case–control studies, and observational studies published in peer-reviewed journals. Only studies published in English were included.

### 2.3 Study selection

The selection of studies involved screening titles and abstracts based on the established eligibility criteria. Full-text articles were subsequently assessed for inclusion. We manually selected the articles for inclusion and collected information on study characteristics, the authors, year of publication, study design.

### 2.4 Data extraction

Details of the patient number, comparison methods (MFC/qRT-PCR), end of induction MRD value and detection thresholds, event free survival rate (EFS), overall survival rate (OS), statistical correlation between NGS-MRD and EFS, OS were extracted from the selected studies. The results were synthesized using a graphic and narrative approach.

### 2.5 PRISMA compliance

This review adheres to PRISMA guidelines, and the PRISMA flow diagram is included to illustrate the study selection process.

## 3 Results

### 3.1 Study selection

**Figure 1:**
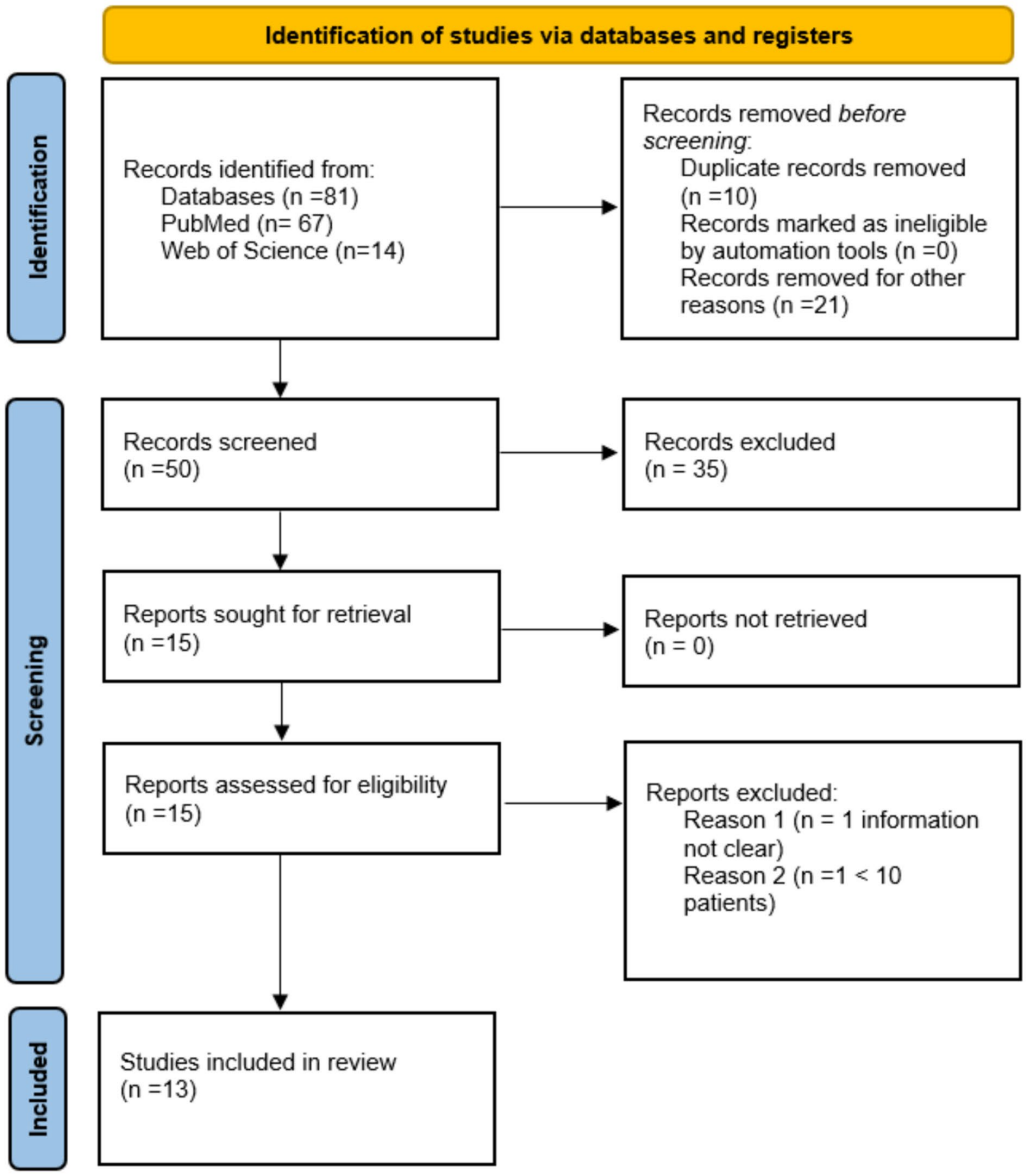
The PRISMA flow diagram.

Following the PRISMA guidelines, a comprehensive literature search was conducted, yielding a total of 81 articles. After screening titles and abstracts, assessing full-text articles, removing duplicates, 13 studies met the eligibility criteria and were included in this review. The PRISMA flow diagram illustrating the study selection process is shown in Figure 1.

### 3.2 Study characteristics

Table 2 summarizes the key characteristics of the included studies, the authors, publication year, study design, sample size, methods used, patient number, ALL phenotype, probe source, end of induction MRD, EFS, OS.

**Table 2:** Summary of key information from reviewed articles on NGS in acute lymphoblastic leukemia.

Study	Design	Total number of patients	Age	Cell phenotype	MRD source	Comparison method	EOI negative 0.01% NGS-MRD patients	EFS 5 y NGS-MRD patients <0.01% EOI	Median observation time
Wu et al. (14)	PCS	99	Pediatric	B cell	BM	MFC	40	NA	NA
Pulsipher et al. (15)	RCS	56	1-21y	B cell	BM	MFC	47	87% (2y LFS)	26 m
Sekiya et al. (16)	RCS	79	Pediatric	B cell	BM	No	27	82.4% (LFS)	76 m
Wood et al. (17)	RCS	619	Pediatric	B cell	BM	MFC	433	>95%	72 m
Jo et al. 2018 (18)	PCS	47	Pediatric + adults	B cell	BM	MFC/qRT-PCR Ig rearrangements	47	100%	139 m
Lu et al. 2021 (19)	PCS	22	Pediatric	B cell	BM	MFC	21	NA	10.5 m
Paolino^†^ et al. 2021 (20)	PCS	317	1–21 y	B/T cell	BM	MFC	NA	NA	NA
Lee et al. (21)	RCS	55	Pediatric	B cell	BM	No	27	100% (3 y EFS)	60 m
Pulsipher^†^ et al. (22)	PCS	143	Pediatric + adults	B cell	BM	MFC	57(10^−6^) *	68% (2y LFS)	38.4 m
Mai^†^ et al. (23)	PCS	64	Pediatric	B/T cell	BM	MFC/qRT-PCR fusion genes	27	NA	NA
Chen et al. (24)	PCS	430	Pediatric	B cell	BM	MFC	125 (IGH)	96% (3y EFS)	20.7 m
Roy et al. (25)	RCS	298 (samples)	Pediatric	T cell	NA	MFC	83	95.20%	60 m
Hwang et al. (26)	PCS	54	Pediatric	B cell	BM	MFC	NA	NA	14.3 m

* Day 28 after Tisagenlecleucel infusion. †Without follow up. EFS, event free survival rate; LFS, leukemia free survival rate; EOI, end of induction; NGS, next generation sequencing; MFC, multiparametric flow cytometry; qRT-PCR, real-time quantitative polymerase chain reaction-RCS, retrospective cohort study; PCS, prospective cohort study; NA, not available; BM, bone marrow; PB, peripheral blood; IGH, immunoglobulin heavy chain locus rearrangement; m, months.

### 3.3 Sensitivity and specificity of NGS in MRD detection

In all included studies, NGS demonstrated high sensitivity for detecting MRD. At the 0.01% threshold, NGS identified a greater number of MRD-positive patients than MFC, highlighting the better sensitivity of NGS in detecting low levels of MRD.

Mai et al. detected more NGS-MRD-positive cases compared to MFC in both B-ALL (57.5% vs. 26.9%) and T-ALL (80% vs. 46.7%), highlighting NGS’s greater sensitivity. Concordance between NGS and MFC was 97.2% for positive MRD and 57.1% for negative MRD. NGS identified MRD-positive cases that where not detectable by MFC. In B-ALL, two or more clonal rearrangements at diagnosis were a significant risk factor for persistent MRD at end of induction. The B-ALL patients had traceable clonal rearrangements (87.9%), with IGH being the most common. When comparing NGS to qRT-PCR for fusion genes, NGS detected more positive samples (52.1% vs. 18.8%) and showed good correlation (*r* = 0.618) (23).

Hwang et al. (26) compared MRD detection using MFC and NGS in pediatric B-ALL patients, showing a concordance rate of 79.9% between the methods. NGS-MRD detected more positive patients, identifying a present MRD in 39.6% of samples compared to 23.7% by MFC-MRD. NGS-MRD had superior sensitivity, detecting low-level MRD in 18% of cases that were negative by MFC, with a median MRD value of 0.0012%. Only 2.2% of cases were positive by MFC, but negative by NGS. Factors such as hemodilution, the presence of hematogones, and evolving leukemic phenotypes impacted MFC performance, while NGS was less affected (26).

### 3.4 Multiparametric flow cytometry and NGS in MRD monitoring

MFC specifically selects viable cells for analysis by identifying surface and intracellular markers. In contrast, NGS relies on DNA quantification for cell enumeration, which can include DNA from both viable and non-viable cells, potentially overestimating the residual leukemic burden. MFC excels in identifying clones not trackable by NGS due to a lack of defined genetic markers. Paolino et al. (20) showed a high correlation between the two methods for patients with detectable MRD (Pearson *r* = 0.87, *p* < 0.0001), suggesting that both assays are generally consistent within their shared sensitivity range. Among patients classified as high MRD at end of induction, 43% (30/70) of the B-ALL patients were identified as high MRD (≥10^−4^) by NGS alone, with the majority near the MFC detection limit (10^-4^). Among T-ALL patients, 75% (21/28) were identified as high MRD by NGS alone, with 67% (14/21) in the range of 10^−4^, and the remaining 33% (7/21) in the higher range of 10^−3^ to <10^−1^. These findings highlight the superior sensitivity of NGS in detecting low-level MRD, particularly for T-ALL, which poses greater challenges for MFC due to immunophenotypic overlap with normal T cells and changes in the clone phenotype after chemotherapy.

### 3.5 Immunoglobulin gene rearrangements

The prognostic value of immunoglobulin heavy chain locus (IGH) rearrangements in MRD monitoring for pediatric B-ALL is established, but the contribution of light chain loci (IGK/IGL) remains unclear. In 2023, Chen et al. aimed to assess their role in evaluating MRD in B-ALL. They discovered that IGK/IGL rearrangements identified 5.5% of patients without detectable IGH clones. Concordance rates for IGH and IGK/IGL MRD detection are 79.9% at the end of induction and 81.0% at the end of consolidation. Patients with NGS-MRD < 0.01% at end of induction (EOI) or < 0.0001% at EOC (end of consolidation) have an excellent prognosis with 3-year event-free survival rates over 95%. IGH rearrangements are prognostic at both EOI and EOC, while IGK and IGL rearrangements provide limited additional prognostic information (24).

### 3.6 Clinical utility of NGS in MRD monitoring

Wood et al. (17) reported not only that NGS, but also that the subgroup etc. has a comparable performance to MFC for identification of poor risk patients at EOI, but also found that the subgroup of patients who were MRD-negative on MFC, but positive on NGS had a poorer prognosis. They also demonstrated that NGS-MRD is an independent prognostic factor in the standard risk group and that patients without a trackable IGH rearrangement have a worse outcome (17).

Pulsipher et al. (15) and Pulsipher et al. (22) demonstrated in two separate studies the utility of NGS-MRD evaluation pre and post hematopoietic stem cell transplantation and cellular therapy with Tisagenlecleucel. NGS-MRD detection prior to and after hematopoietic cell transplantation in patients with ALL is more predictive of relapse and survival than MFC. NGS-MRD showed a significantly lower relapse rate (0% vs. 16%) and higher overall survival (96% vs. 77%) compared to MFC-MRD, in MRD-negative patients, highlighting its potential for guiding treatment intensity and early post-HCT interventions (15). In the cellular therapy with Tisagenlecleucel study, the data demonstrated that NGS-MRD from the bone marrow represents a very sensitive biomarker for predicting relapse risk following CAR-T cell therapy, particularly at a sensitivity threshold as low as 10^−6^. At day 28 post-infusion, the prognostic significance of NGS-MRD was found when any level of detectable disease was classified as high risk. Importantly, by 3 months and at all subsequent time points up to 12 months, the presence of detectable NGS-MRD was highly prognostic, with 41 out of 42 patients exhibiting measurable disease either relapsing or being censored due to hematopoietic cell transplant or other therapies (22).

### 3.7 Clonal evolution

#### 3.7.1 Clonal evolution during induction chemotherapy

The data from Wu et al. study showed that, among the pretreatment samples in which variable heavy chain (V_H_)-replaced clones were detected, 0.4% (median) were evolved clonotypes of the total clone. Throughout the induction therapy there was minimal change in the relative proportions of the dominant clone and V_H_-replaced subclones. On average, 0.036% of IGH rearrangements were consistent with V_H_ replacement of the major clone after treatment. Combing the information with the MRD results, the authors concluded that within early time from diagnosis, ongoing rearrangements at the IGH locus does not significantly change the response of lymphoblast clones during initial treatment (14).

#### 3.7.2 Clonal evolution at relapse

Sekiya et al. (16) identified changes in complementarity-determining region 3 (CDR3) sequences between initial diagnosis and relapse. In 86% of patients, the same CDR3 sequences detected at diagnosis were present at relapse. Clonal evolution was observed in 3/15 relapsed patients, with new subclones appearing at relapse.

## 4 Discussions

Next generation sequencing, has emerged as a transformative tool for MRD monitoring in ALL, addressing several limitations associated with traditional methods such as MFC and qRT-PCR. The reviewed studies highlight the superior sensitivity of NGS. This high sensitivity is particularly valuable in identifying low-level MRD in patients who are classified as MRD-negative by MFC (17). The inclusion of IGH rearrangements as the primary marker in NGS panels has demonstrated good prognostic value in B-ALL, with patients achieving MRD-negative status by NGS showing significantly higher EFS and OS rates. While IGH remains the cornerstone for MRD monitoring, the inclusion of additional loci such as IGK and IGL has proven useful in identifying MRD-positive patients who lack detectable IGH clones, although their independent prognostic contribution is limited (24).

NGS has shown good effectiveness in determining a picture of clonal IGH evolution. Using high-resolution sequencing, Gawad et al. (27) provided valuable insights into the molecular mechanisms driving clonal evolution in B cell ALL. Their results suggested that V_H_ replacement is the main mechanism contributing to clonal evolution. In other studies NGS showed that the dominant clone found in relapsed patients was the same as the one from diagnosis, suggesting that an important factor for relapse is the acquisition of chemoresistance to the initial therapy (14, 16).

Another beneficial aspect of NGS is that, due to its higher sensitivity, MRD assessment can be performed using peripheral blood, provided that sufficient DNA is available, which is significantly less traumatic than bone marrow aspiration, particularly for the pediatric population.

Beyond sensitivity, the clinical utility of NGS extends to its predictive power for relapse and survival across various therapeutic contexts, including hematopoietic stem cell transplantation and CAR-T cell therapy (15, 22).

Despite these advantages, several challenges must be addressed to fully integrate NGS into routine clinical practice. The method’s reliance on DNA sequencing, while enhancing sensitivity, can lead to overestimation of MRD due to the inclusion of DNA from non-viable cells. Another important aspect is the distinction of leukemia-specific IGH or TRG rearrangements from the normal background. In 2014, Wu et al. proposed a threshold of >10% of nucleated cells for defining leukemia-specific sequences. In the Children’s Oncology Group ALL00932 trial he and his colleagues identified leukemia-specific sequences in 92 out of 99 (93%) patients based on this definition (14). Alternatively, other studies used a lower threshold of 5% (11, 12, 15, 17). High costs, the complexity of bioinformatics analysis and variability in reporting standards further restrict its widespread adoption. Standardizing NGS protocols and establishing clinically validated thresholds for MRD detection are essential steps for ensuring its broader applicability (13, 28).

The ability of NGS to identify residual disease that is undetectable by other methods offers opportunities to escalate treatment intensity for high-risk patients or de-escalate therapy for low-risk individuals, minimizing the risk of relapse while reducing treatment-related toxicity. Combining MFC and NGS could offer complementary benefits. MFC can identify viable MRD-positive cells based on phenotype and NGS provides deeper sensitivity to detect subclones or low-level MRD below MFC threshold. Advances in both fields, such as integrating MFC with molecular markers or enhancing NGS panel diversity, aim to refine their combined utility in therapeutic decision-making (29, 30).

## 5 Conclusion

NGS is redefining the standard of care for MRD monitoring in ALL by offering additional sensitivity and prognostic accuracy. However, its widespread implementation requires further research into cost-effectiveness, protocol standardization and its integration with emerging therapies. Future studies should focus on expanding NGS panels, improving the bioinformatic algorithms for MRD quantification and evaluating its impact in various clinical settings. By addressing these challenges, NGS has the potential to become the gold standard for MRD monitoring, significantly advancing personalized medicine in ALL.

## Data Availability

The original contributions presented in the study are included in the article/Supplementary material, further inquiries can be directed to the corresponding author.
