# Supplementary material for: The emerging role of next-generation sequencing in minimal residual disease assessment in acute lymphoblastic leukemia: a systematic review of current literature
Source: Front Med (Lausanne). 2025 Apr 22;12:1570041. doi: 10.3389/fmed.2025.1570041 (PMC12052730; doi:10.3389/fmed.2025.1570041)
Supplement: Supplementary file 1 [file Data_Sheet_1.pdf]

The emerging role of next-generation sequencing in minimal residual disease assessment in acute lymphoblastic leukemia: a systematic review of current literature

1. Background

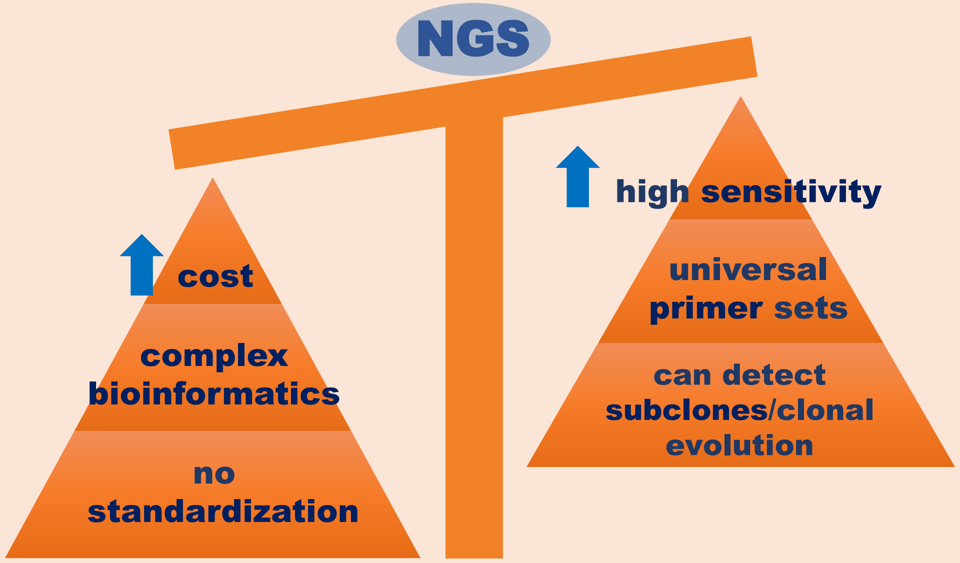

2. Objective

- evaluation of clinical utility and prognostic value of NGS for MRD detection in ALL vs MFC and qRT-PCR
- provide a comprehensive analysis of recent studies

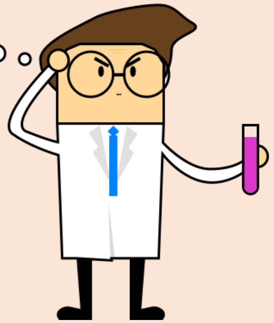

3. Methods

Inclusion criteria:

- Diagnostic - ALL
- MRD evaluation - NGS
- Minimum patients – 10
- Language – English

PubMed® + Web of Science™

4. Results

= 13 papers

NGS

- good correlation with outcome
- predicted relapse
- better sensitivity

5. Discussions

- NGS improves prognostic accuracy
- standardization and cost remain challenges

ALL- acute lymphoblastic leukemia
MFC- multiparametric flow cytometry
qRT-PCR- real-time quantitative PCR
